# *N*-(2,3-Dihydro-1,3-thia­zol-2-yl­idene)-4-[(2-hydroxy­benzyl­idene)amino]benzene­sulfonamide

**DOI:** 10.1107/S1600536809033704

**Published:** 2009-08-29

**Authors:** Xin-Li Zhang

**Affiliations:** a Department of Chemistry, Baoji University of Arts and Sciences, Baoji, Shaanxi 721007, People’s Republic of China

## Abstract

The title compound, C_16_H_13_N_3_O_3_S_2_, was prepared by reaction of salicylaldehyde and sulfathia­zole in methanol. The dihedral angle between the central benzene ring and the thia­zole ring is 85.2 (2)° and that between the two benzene rings is 17.9 (2)°. An intra­molecular O—H⋯N hydrogen bond generates an *S*(6) ring motif. In the crystal, mol­ecules are held together by inter­molecular N—H⋯N and C—H⋯O hydrogen bonds, forming a two-dimensional network parallel to the *bc* plane.

## Related literature

For the biological activity of Schiff bases, see: Billson *et al.* (2000 ▶); Carlton *et al.* (1995 ▶). For a related structure, see: Li *et al.* (2006 ▶).
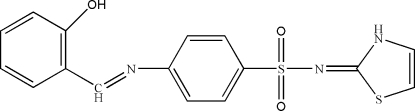


## Experimental

### 

#### Crystal data

C_16_H_13_N_3_O_3_S_2_
*M* *_r_* = 359.41
Monoclinic, 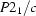

*a* = 16.1693 (18) Å
*b* = 9.1211 (11) Å
*c* = 11.0292 (13) Å
β = 101.896 (1)°
*V* = 1591.7 (3) Å^3^
*Z* = 4
Mo *K*α radiation
μ = 0.36 mm^−1^
*T* = 298 K
0.40 × 0.37 × 0.20 mm

#### Data collection

Bruker SMART CCD area-detector diffractometer
Absorption correction: multi-scan (*SADABS*; Sheldrick, 1996 ▶) *T*_min_ = 0.871, *T*_max_ = 0.932
7737 measured reflections
2806 independent reflections
1927 reflections with *I* > 2σ(*I*)
*R*_int_ = 0.031

#### Refinement

*R*[*F* ^2^ > 2σ(*F* ^2^)] = 0.041
*wR*(*F* ^2^) = 0.108
*S* = 1.04
2806 reflections
224 parameters
H atoms treated by a mixture of independent and constrained refinement
Δρ_max_ = 0.30 e Å^−3^
Δρ_min_ = −0.30 e Å^−3^

### 

Data collection: *SMART* (Bruker, 2000 ▶); cell refinement: *SAINT* (Bruker, 2000 ▶); data reduction: *SAINT*; program(s) used to solve structure: *SHELXS97* (Sheldrick, 2008 ▶); program(s) used to refine structure: *SHELXL97* (Sheldrick, 2008 ▶); molecular graphics: *SHELXTL* (Sheldrick, 2008 ▶); software used to prepare material for publication: *SHELXTL*.

## Supplementary Material

Crystal structure: contains datablocks I, global. DOI: 10.1107/S1600536809033704/ci2883sup1.cif

Structure factors: contains datablocks I. DOI: 10.1107/S1600536809033704/ci2883Isup2.hkl

Additional supplementary materials:  crystallographic information; 3D view; checkCIF report

## Figures and Tables

**Table 1:** Hydrogen-bond geometry (Å, °)

*D*—H⋯*A*	*D*—H	H⋯*A*	*D*⋯*A*	*D*—H⋯*A*
O3—H3*A*⋯N1	0.97 (4)	1.73 (4)	2.636 (4)	154 (4)
N2—H2*A*⋯N3^i^	0.89 (3)	1.97 (3)	2.856 (3)	179 (3)
C6—H6⋯O1^ii^	0.93	2.58	3.334 (4)	139
C16—H16⋯O2^iii^	0.93	2.52	3.351 (4)	148

Symmetry codes: (i) 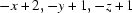; (ii) 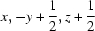; (iii) 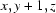.

**Fig. 1.**
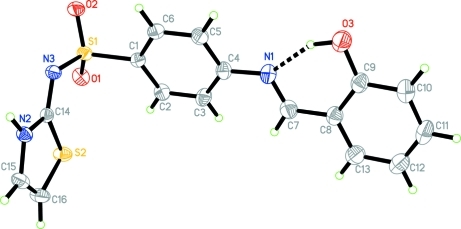
The molecular structure of (I), showing 30% probability displacement ellipsoids. Hydrogen bonds are shown as dashed lines.

**Fig. 2.**
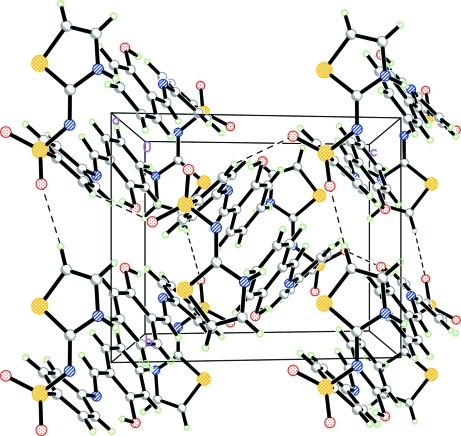
The crystal packing of (I), viewed along the b axis. Hydrogen bonds are shown as dashed lines.

## supplementary crystallographic information

### Comment

The synthesis and characterization of Schiff base compounds have received a great deal of attention due to their biological activities, such as anti-bacterial, anti-cancer and anti-virus (Billson *et al.*, 2000; Carlton *et al.*, 1995). In this paper, we report the crystal structure of the title compound, a new Schiff-base ligand, (I).

Bbond lengths and angles in (I) are normal and they agree with those observed in a salicylaldehyde Schiff base (Li *et al.*, 2006). The C7db// N1 bond length of 1.269 (4) Å conforms to the  value for a double bond. The dihedral angle between C1-C6 benzene ring and thiazole ring is 85.2 (2)°. The dihedral angle between the two benzene rings is 17.9 (2)°. An intramolecular O3—H3A···N1 hydrogen bond generates an S(6) ring motif (Fig. 1).

The molecules are held together by N—H···N and C—H···O intermolecular hydrogen bonds (Table 1), forming a two-dimensional network parallel to the *bc* plane (Fig. 2).

### Experimental

All chemicals were of reagent grade and commercially available from the Beijing Chemical Reagents Company of China, and were used without further purification. A methanol solution (10 ml) of salicylaldehyde (0.1 mmol, 12.2 mg) and sulfathiazloe (0.1 mmol, 25.5 mg) was stirred at room temperature for 30 min and then filtered. The filtrate was left to stand in air for 7 d, and the title compound was formed in slow evaporation of the solvent. The crystals were collected, washed with methanol and dried in a vacuum desiccator using anhydrous CaCl_2_ (yield 64%).

### Refinement

Atoms H2A and H3A were located in a difference map and refined freely. The remaining H atoms were positioned geometrically [C-H = 0.93 Å] and constrained to ride on their parent atoms, with *U*_iso_(H) = 1.2*_U_*_eq_(C).

### Crystal data

**Table:**

C_16_H_13_N_3_O_3_S_2_	*F*(000) = 744
*M**_r_* = 359.41	*D*_x_ = 1.500 Mg m^−^^3^
Monoclinic, *P*2_1_/*c*	Mo *K*α radiation, λ = 0.71073 Å
Hall symbol: -P 2ybc	Cell parameters from 2137 reflections
*a* = 16.1693 (18) Å	θ = 2.6–25.0°
*b* = 9.1211 (11) Å	µ = 0.36 mm^−^^1^
*c* = 11.0292 (13) Å	*T* = 298 K
β = 101.896 (1)°	Block, yellow
*V* = 1591.7 (3) Å^3^	0.40 × 0.37 × 0.20 mm
*Z* = 4	

### Data collection

**Table:**

Bruker SMART CCD area-detector diffractometer	2806 independent reflections
Radiation source: fine-focus sealed tube	1927 reflections with *I* > 2σ(*I*)
graphite	*R*_int_ = 0.031
φ and ω scans	θ_max_ = 25.0°, θ_min_ = 1.3°
Absorption correction: multi-scan (*SADABS*; Sheldrick, 1996)	*h* = −19→19
*T*_min_ = 0.871, *T*_max_ = 0.932	*k* = −10→10
7737 measured reflections	*l* = −8→13

### Refinement

**Table:**

Refinement on *F*^2^	Primary atom site location: structure-invariant direct methods
Least-squares matrix: full	Secondary atom site location: difference Fourier map
*R*[*F*^2^ > 2σ(*F*^2^)] = 0.041	Hydrogen site location: inferred from neighbouring sites
*wR*(*F*^2^) = 0.108	H atoms treated by a mixture of independent and constrained refinement
*S* = 1.04	*w* = 1/[σ^2^(*F*_o_^2^) + (0.04*P*)^2^ + 1.0601*P*] where *P* = (*F*_o_^2^ + 2*F*_c_^2^)/3
2806 reflections	(Δ/σ)_max_ = 0.001
224 parameters	Δρ_max_ = 0.30 e Å^−^^3^
0 restraints	Δρ_min_ = −0.30 e Å^−^^3^

### Special details

**Table:**

Geometry. All e.s.d.'s (except the e.s.d. in the dihedral angle between two l.s. planes) are estimated using the full covariance matrix. The cell e.s.d.'s are taken into account individually in the estimation of e.s.d.'s in distances, angles and torsion angles; correlations between e.s.d.'s in cell parameters are only used when they are defined by crystal symmetry. An approximate (isotropic) treatment of cell e.s.d.'s is used for estimating e.s.d.'s involving l.s. planes.
Refinement. Refinement of *F*^2^ against ALL reflections. The weighted *R*-factor *wR* and goodness of fit *S* are based on *F*^2^, conventional *R*-factors *R* are based on *F*, with *F* set to zero for negative *F*^2^. The threshold expression of *F*^2^ > σ(*F*^2^) is used only for calculating *R*-factors(gt) *etc*. and is not relevant to the choice of reflections for refinement. *R*-factors based on *F*^2^ are statistically about twice as large as those based on *F*, and *R*- factors based on ALL data will be even larger.

### Fractional atomic coordinates and isotropic or equivalent isotropic displacement parameters (Å^2^)

**Table:**

	*x*	*y*	*z*	*U*_iso_*/*U*_eq_	
N1	0.54316 (16)	0.2823 (3)	0.3783 (2)	0.0517 (7)	
N2	0.95632 (15)	0.6826 (3)	0.4499 (2)	0.0375 (6)	
H2A	0.9910 (19)	0.640 (3)	0.513 (3)	0.055 (10)*	
N3	0.93153 (14)	0.4585 (2)	0.3503 (2)	0.0370 (6)	
O1	0.86589 (13)	0.4333 (2)	0.12312 (17)	0.0484 (5)	
O2	0.91746 (13)	0.2184 (2)	0.2533 (2)	0.0506 (6)	
O3	0.45423 (16)	0.1377 (3)	0.5144 (2)	0.0728 (8)	
H3A	0.498 (3)	0.166 (5)	0.470 (4)	0.109*	
S1	0.87791 (4)	0.35993 (8)	0.24101 (7)	0.0379 (2)	
S2	0.86729 (5)	0.71804 (9)	0.23688 (7)	0.0475 (2)	
C1	0.77728 (17)	0.3381 (3)	0.2773 (3)	0.0363 (7)	
C2	0.70885 (18)	0.4196 (3)	0.2163 (3)	0.0441 (8)	
H2	0.7157	0.4862	0.1553	0.053*	
C3	0.63071 (18)	0.4019 (4)	0.2458 (3)	0.0494 (8)	
H3B	0.5849	0.4559	0.2036	0.059*	
C4	0.61981 (19)	0.3047 (4)	0.3375 (3)	0.0458 (8)	
C5	0.68850 (19)	0.2214 (3)	0.3959 (3)	0.0482 (8)	
H5	0.6814	0.1526	0.4551	0.058*	
C6	0.76694 (19)	0.2390 (3)	0.3675 (3)	0.0438 (7)	
H6	0.8127	0.1845	0.4090	0.053*	
C7	0.4851 (2)	0.3782 (4)	0.3608 (3)	0.0555 (9)	
H7	0.4919	0.4613	0.3151	0.067*	
C8	0.40865 (19)	0.3643 (4)	0.4087 (3)	0.0496 (8)	
C9	0.39700 (19)	0.2481 (4)	0.4865 (3)	0.0527 (8)	
C10	0.3252 (2)	0.2432 (4)	0.5367 (3)	0.0633 (10)	
H10	0.3174	0.1666	0.5889	0.076*	
C11	0.2658 (2)	0.3519 (5)	0.5089 (3)	0.0662 (11)	
H11	0.2180	0.3485	0.5435	0.079*	
C12	0.2749 (2)	0.4648 (5)	0.4319 (4)	0.0655 (10)	
H12	0.2333	0.5364	0.4125	0.079*	
C13	0.3464 (2)	0.4713 (4)	0.3833 (3)	0.0625 (10)	
H13	0.3534	0.5493	0.3320	0.075*	
C14	0.92217 (16)	0.6028 (3)	0.3501 (2)	0.0336 (6)	
C15	0.9408 (2)	0.8303 (3)	0.4400 (3)	0.0477 (8)	
H15	0.9614	0.8969	0.5029	0.057*	
C16	0.8939 (2)	0.8685 (4)	0.3323 (3)	0.0554 (9)	
H16	0.8772	0.9641	0.3104	0.066*	

### Atomic displacement parameters (Å^2^)

**Table:**

	*U*^11^	*U*^22^	*U*^33^	*U*^12^	*U*^13^	*U*^23^
N1	0.0409 (15)	0.0615 (19)	0.0528 (17)	−0.0026 (14)	0.0101 (13)	0.0003 (14)
N2	0.0399 (14)	0.0344 (14)	0.0362 (14)	−0.0024 (11)	0.0033 (12)	−0.0005 (12)
N3	0.0402 (13)	0.0306 (14)	0.0378 (13)	0.0001 (11)	0.0027 (11)	−0.0001 (11)
O1	0.0591 (13)	0.0538 (14)	0.0323 (11)	−0.0072 (11)	0.0092 (9)	−0.0014 (10)
O2	0.0532 (13)	0.0349 (12)	0.0674 (15)	0.0032 (10)	0.0213 (11)	−0.0078 (11)
O3	0.0607 (16)	0.0759 (19)	0.0865 (19)	0.0096 (14)	0.0256 (14)	0.0188 (15)
S1	0.0420 (4)	0.0339 (4)	0.0378 (4)	−0.0013 (3)	0.0086 (3)	−0.0048 (3)
S2	0.0595 (5)	0.0377 (5)	0.0424 (5)	0.0064 (4)	0.0033 (4)	0.0063 (4)
C1	0.0392 (16)	0.0360 (16)	0.0320 (15)	−0.0005 (13)	0.0032 (12)	−0.0047 (13)
C2	0.0451 (18)	0.0501 (19)	0.0344 (16)	−0.0025 (15)	0.0018 (14)	0.0069 (15)
C3	0.0388 (17)	0.062 (2)	0.0437 (19)	0.0040 (15)	0.0005 (14)	0.0058 (16)
C4	0.0422 (18)	0.052 (2)	0.0427 (18)	−0.0049 (15)	0.0083 (14)	−0.0030 (15)
C5	0.0508 (19)	0.048 (2)	0.0475 (19)	−0.0005 (16)	0.0134 (15)	0.0095 (15)
C6	0.0452 (18)	0.0428 (18)	0.0427 (18)	0.0046 (14)	0.0073 (14)	0.0029 (14)
C7	0.054 (2)	0.057 (2)	0.057 (2)	−0.0035 (18)	0.0133 (17)	0.0027 (17)
C8	0.0437 (18)	0.056 (2)	0.0481 (19)	−0.0037 (16)	0.0083 (15)	−0.0077 (17)
C9	0.0405 (18)	0.062 (2)	0.053 (2)	−0.0013 (17)	0.0044 (16)	−0.0071 (18)
C10	0.054 (2)	0.078 (3)	0.060 (2)	−0.011 (2)	0.0173 (18)	−0.007 (2)
C11	0.045 (2)	0.092 (3)	0.064 (2)	−0.004 (2)	0.0176 (18)	−0.025 (2)
C12	0.052 (2)	0.072 (3)	0.070 (3)	0.0065 (19)	0.0075 (19)	−0.019 (2)
C13	0.058 (2)	0.062 (2)	0.066 (2)	0.0037 (19)	0.0111 (19)	−0.0043 (19)
C14	0.0300 (14)	0.0360 (17)	0.0356 (16)	−0.0015 (12)	0.0087 (12)	−0.0014 (13)
C15	0.056 (2)	0.0333 (18)	0.054 (2)	−0.0060 (15)	0.0122 (16)	−0.0056 (15)
C16	0.068 (2)	0.0320 (18)	0.066 (2)	0.0033 (16)	0.0122 (19)	0.0023 (16)

### Geometric parameters (Å, °)

**Table:**

N1—C7	1.269 (4)	C4—C5	1.389 (4)
N1—C4	1.418 (4)	C5—C6	1.377 (4)
N2—C14	1.340 (3)	C5—H5	0.93
N2—C15	1.371 (4)	C6—H6	0.93
N2—H2A	0.89 (3)	C7—C8	1.446 (4)
N3—C14	1.325 (3)	C7—H7	0.93
N3—S1	1.607 (2)	C8—C13	1.388 (4)
O1—S1	1.440 (2)	C8—C9	1.401 (5)
O2—S1	1.434 (2)	C9—C10	1.386 (5)
O3—C9	1.359 (4)	C10—C11	1.371 (5)
O3—H3A	0.97 (4)	C10—H10	0.93
S1—C1	1.765 (3)	C11—C12	1.362 (5)
S2—C16	1.728 (3)	C11—H11	0.93
S2—C14	1.730 (3)	C12—C13	1.373 (5)
C1—C6	1.379 (4)	C12—H12	0.93
C1—C2	1.387 (4)	C13—H13	0.93
C2—C3	1.377 (4)	C15—C16	1.318 (4)
C2—H2	0.93	C15—H15	0.93
C3—C4	1.384 (4)	C16—H16	0.93
C3—H3B	0.93		
			
C7—N1—C4	121.4 (3)	N1—C7—C8	123.1 (3)
C14—N2—C15	115.6 (3)	N1—C7—H7	118.5
C14—N2—H2A	119 (2)	C8—C7—H7	118.5
C15—N2—H2A	125 (2)	C13—C8—C9	118.2 (3)
C14—N3—S1	120.72 (19)	C13—C8—C7	120.2 (3)
C9—O3—H3A	103 (3)	C9—C8—C7	121.5 (3)
O2—S1—O1	118.59 (13)	O3—C9—C10	118.2 (3)
O2—S1—N3	105.74 (12)	O3—C9—C8	121.9 (3)
O1—S1—N3	111.64 (12)	C10—C9—C8	119.9 (3)
O2—S1—C1	106.91 (13)	C11—C10—C9	119.6 (4)
O1—S1—C1	107.36 (13)	C11—C10—H10	120.2
N3—S1—C1	105.84 (12)	C9—C10—H10	120.2
C16—S2—C14	90.97 (15)	C12—C11—C10	121.7 (4)
C6—C1—C2	119.9 (3)	C12—C11—H11	119.2
C6—C1—S1	119.5 (2)	C10—C11—H11	119.2
C2—C1—S1	120.7 (2)	C11—C12—C13	118.9 (4)
C3—C2—C1	120.1 (3)	C11—C12—H12	120.5
C3—C2—H2	119.9	C13—C12—H12	120.5
C1—C2—H2	119.9	C12—C13—C8	121.7 (4)
C2—C3—C4	120.6 (3)	C12—C13—H13	119.2
C2—C3—H3B	119.7	C8—C13—H13	119.2
C4—C3—H3B	119.7	N3—C14—N2	120.7 (3)
C3—C4—C5	118.6 (3)	N3—C14—S2	130.3 (2)
C3—C4—N1	125.2 (3)	N2—C14—S2	109.0 (2)
C5—C4—N1	116.2 (3)	C16—C15—N2	113.1 (3)
C6—C5—C4	121.1 (3)	C16—C15—H15	123.4
C6—C5—H5	119.4	N2—C15—H15	123.4
C4—C5—H5	119.4	C15—C16—S2	111.3 (2)
C5—C6—C1	119.6 (3)	C15—C16—H16	124.3
C5—C6—H6	120.2	S2—C16—H16	124.3
C1—C6—H6	120.2		
			
C14—N3—S1—O2	166.3 (2)	N1—C7—C8—C13	−178.2 (3)
C14—N3—S1—O1	36.0 (3)	N1—C7—C8—C9	5.1 (5)
C14—N3—S1—C1	−80.5 (2)	C13—C8—C9—O3	178.9 (3)
O2—S1—C1—C6	34.9 (3)	C7—C8—C9—O3	−4.3 (5)
O1—S1—C1—C6	163.2 (2)	C13—C8—C9—C10	−0.7 (5)
N3—S1—C1—C6	−77.5 (3)	C7—C8—C9—C10	176.1 (3)
O2—S1—C1—C2	−145.2 (2)	O3—C9—C10—C11	−179.1 (3)
O1—S1—C1—C2	−16.9 (3)	C8—C9—C10—C11	0.5 (5)
N3—S1—C1—C2	102.4 (2)	C9—C10—C11—C12	0.6 (5)
C6—C1—C2—C3	0.1 (4)	C10—C11—C12—C13	−1.5 (5)
S1—C1—C2—C3	−179.8 (2)	C11—C12—C13—C8	1.3 (5)
C1—C2—C3—C4	0.9 (5)	C9—C8—C13—C12	−0.2 (5)
C2—C3—C4—C5	−2.4 (5)	C7—C8—C13—C12	−177.0 (3)
C2—C3—C4—N1	177.4 (3)	S1—N3—C14—N2	169.4 (2)
C7—N1—C4—C3	−21.1 (5)	S1—N3—C14—S2	−8.7 (4)
C7—N1—C4—C5	158.7 (3)	C15—N2—C14—N3	−178.5 (3)
C3—C4—C5—C6	2.8 (5)	C15—N2—C14—S2	0.0 (3)
N1—C4—C5—C6	−177.0 (3)	C16—S2—C14—N3	178.1 (3)
C4—C5—C6—C1	−1.8 (5)	C16—S2—C14—N2	−0.1 (2)
C2—C1—C6—C5	0.3 (4)	C14—N2—C15—C16	0.3 (4)
S1—C1—C6—C5	−179.8 (2)	N2—C15—C16—S2	−0.4 (4)
C4—N1—C7—C8	−175.4 (3)	C14—S2—C16—C15	0.3 (3)

### Hydrogen-bond geometry (Å, °)

**Table:**

*D*—H···*A*	*D*—H	H···*A*	*D*···*A*	*D*—H···*A*
O3—H3A···N1	0.97 (4)	1.73 (4)	2.636 (4)	154 (4)
N2—H2A···N3^i^	0.89 (3)	1.97 (3)	2.856 (3)	179 (3)
C6—H6···O1^ii^	0.93	2.58	3.334 (4)	139
C16—H16···O2^iii^	0.93	2.52	3.351 (4)	148

Symmetry codes: (i) −*x*+2, −*y*+1, −*z*+1; (ii) *x*, −*y*+1/2, *z*+1/2; (iii) *x*, *y*+1, *z*.
